# Supplementary material for: Early selection of task-relevant features through population gating
Source: Nat Commun. 2023 Oct 27;14:6837. doi: 10.1038/s41467-023-42519-5 (PMC10603060; doi:10.1038/s41467-023-42519-5)
Supplement: Supplementary file 1 — Supplementary Information [file 41467_2023_42519_MOESM1_ESM.pdf]

Supplementary Material

Supplementary Notes

Discarding motor execution confounds

At the level of the task design, action (go left / go right) and the relevant go stimuli (left / low pitch) were not dissociable, which could in principle confound the abstract representation of the relevant stimuli with activity related to motor execution. We investigated this potential confound in two complementary ways. First, we cross-correlated the decoding weights estimated during different time points across the trial (Fig. S5). These analyses revealed that relevant and irrelevant decoding dimensions were constrained to stimulus presentation and that a third dimension was explored after the stimulus offset. This axis was orthogonal to the aforementioned stimuli axis (Fig. S5), likely encoding the rat's ongoing motor execution. To avoid confounding motor execution with the encoding of relevant stimuli, this period was ignored from all analyses and only the sensory and selection axes — orthogonal to the motor axis — were interpreted. In contrast, PFC did not encode the irrelevant stimulus and the decoding of relevant stimulus was well aligned with decision decoding (Fig. S5), suggesting that the decoding of relevant stimulus from PFC was in fact capturing motor variability. Second, we artificially decorrelated decisions and relevant stimuli by enforcing an equal amount of error and correct pseudo-trials during sampling. We have found that it is possible to decode both relevant stimuli and the choice from A1 and PFC (Fig. S5c). Importantly, in contrast to A1, selecting PFC neurons based on their pre-stimulus modulation to context did not reveal a population structure with output-gating (Methods, Fig. 3), indicating further that grouping A1 neurons by their pre-stimulus firing rate did not select motor related neurons. In a recent experiment (Yin et al. 2020) that decoupled sounds from motor output (i.e. go vs no-go), Yin and colleagues found that sound encoding emerged earlier in the ferret A1 (25 ms) than in PFC (50-100 ms). On the other hand, motor output related information emerged earlier in PFC (50 ms) and feedback information appeared in A1 around 600ms (their Fig. 4), one order of magnitude later than what we here interpret as stimulus encoding ($1-2$ bins, $< 25-50$ ms; Fig. 1,3) but in line with the aforementioned motor-related variability in A1 that we discarded from our analyses (Fig. S5). Ultimately, only task designs decoupling decision and stimulus will be able to determine if decision is already computed in A1 before reaching PFC; notwithstanding, the mechanisms proposed here for stimulus selection would straightforwardly apply to decision selection within A1.

Slight asymmetry between contexts

We found stronger encoding of location than of pitch in A1 (Fig. 1,3), but we are reluctant to interpret this as a general finding. Instead, we speculate that this slight asymmetry between the two contexts was due to all recordings being performed in the left brain hemisphere. Crucially, the noise bursts indicating "nogo" came from the right, contralateral to the recordings and expected to cause stronger responses than stimuli presented on the ipsilateral hemifield. In addition, the animals showed lower performance in pitch blocks (Rodgers and DeWeese 2014). Together, these asymmetries in the task may explain the differences in effect size between the two contexts. Future experiments with perfectly symmetric tasks and/or bilateral recordings are necessary to validate this possibility, but they will not change the qualitative interpretation of our results.

Supplementary Figures

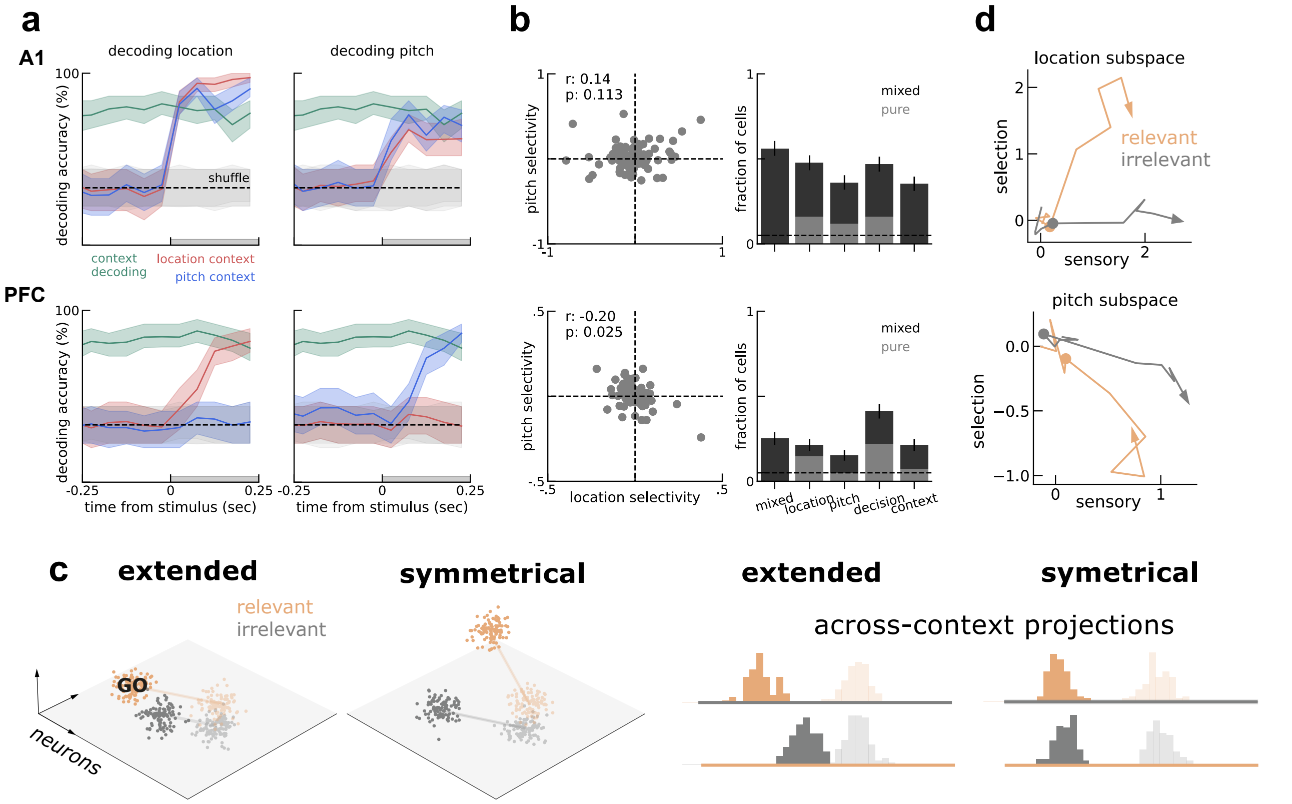

Supplementary Figure 1: *A1 and PFC encode different task variables.* **a)** Logistic regression decoding (Methods) of location (left), pitch (right) in each context (red and blue) and decoding of context overlaid on both plots for comparison. Top, A1 encodes both stimulus' features in either contexts. Bottom, PFC encodes only the relevant stimulus' features for the ongoing context. **b)** Left, feature-selectivity is mixed in both areas. Each axis corresponds to the beta weights corresponding to location and picth selectivity of the linear model (Methods). Each dot is a neuron. Right, fraction of cells with significant task-variables regressors (Methods). Error-bars are bootstrapped SEM (Efron 1981). **c)** The three possible scenarios detailed in the main figure do not provide an exhaustive representation of all possible scenarios. Here we illustrate two other possible scenarios. In the extended scenario, the relevant stimulus is enhanced (similarly to the selected code), but both axes are parallel, so a selection axis does not exist. The symmetrical case is very similar to the selected code. In both scenarios there is a selection axis, but in the selected code the relevant go stimulus encoding is further enhanced. The important message is that the angle between relevant and irrelevant decoding axis and the decoding performance fully characterize the encoding geometry of A1. **d)** Similar analyses to Fig. 1, but forcing the activity during both context to explore the same selection axis. This common axis was determined as the average each context selection axis.

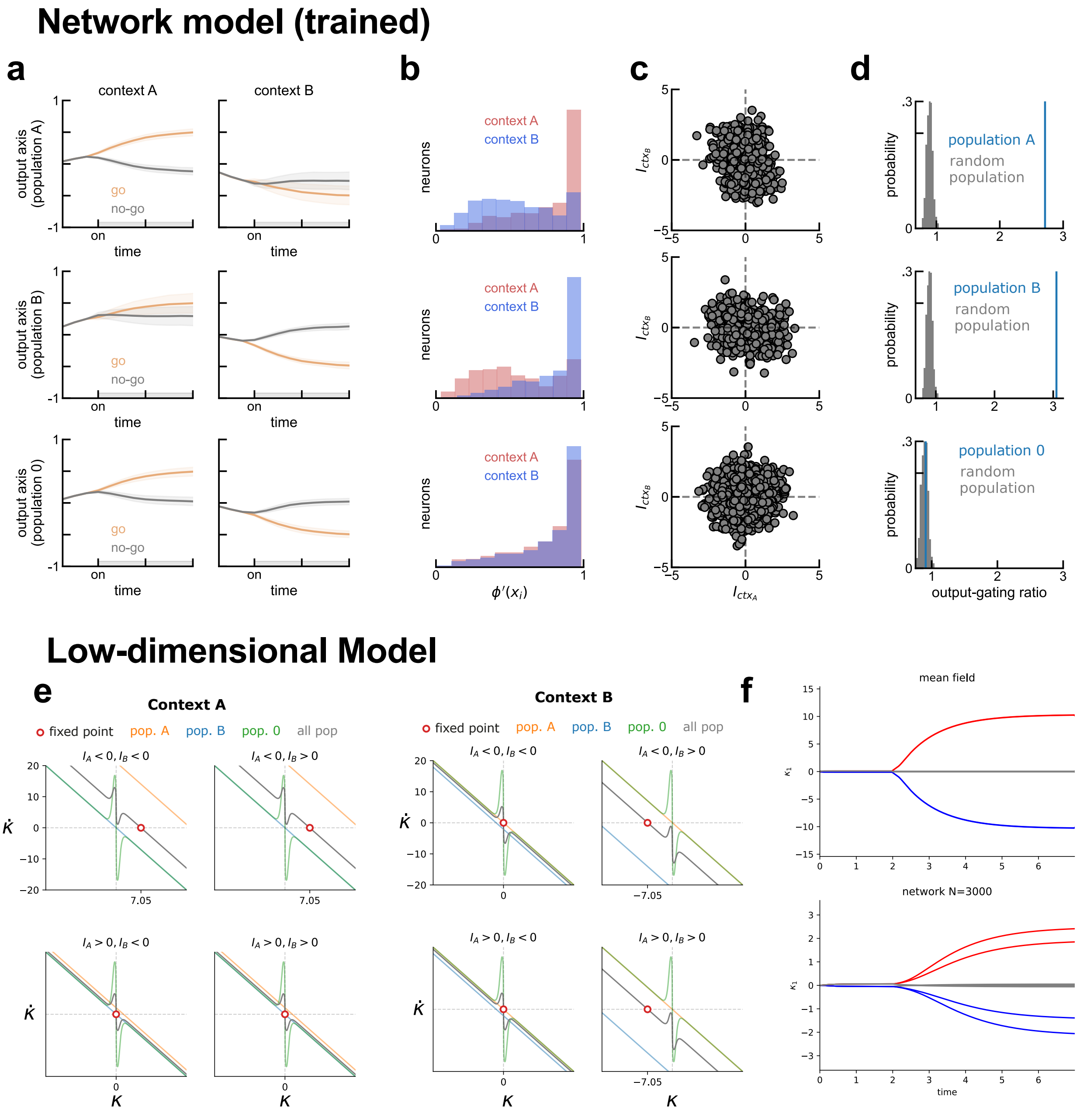

Supplementary Figure 2: *Network and low-dimensional model of a context-dependent go/no-go task.* **a,b)** Same as Fig. 2c and d, but showing data from all three populations. **c)** Context weights optimized through training have different variances for each population, the mechanism supporting gain modulation (Dubreuil et al. 2022). Population A and B have a larger range of weights for context B and A, respectively. Population 0 has the same range of weights for both contextual inputs. **d)** Populations A and B showed substantially more context-dependent activity along the readout axis than population 0 as measured with output-gating ratio (Methods). **e)** Dynamics of kappa separated for each population (color lines) and collectively for all populations (gray) for both contexts and all stimuli combinations. Here it can be seen that population 0 (green) contributes equally for all contexts and stimulus conditions. Namely, it pushes the dynamics of kappa towards 0, essential to have a fixed corresponding to the no go conditions (two bottom conditions for context A, on the left; and two left conditions for context B, on the right). In contrast, population A (orange) and B (blue) are inactive during context B and A, respectively, and do not contribute to the dynamics during those conditions. On the other hand, the same populations are active in opposite contexts and integrate the relevant stimulus into kappa dynamics (e.g. orange lines in context A when input A < 0). **f)** Top, dynamics of the low-dimensional model for all trials. Bottom, dynamics a network with weights sampled from the distribution defined in f (Methods). Here red, gray and blue correspond to "go left", "no go" and "go right", respectively. Quantitative differences due to finite-size effects and reduced when using larger networks.

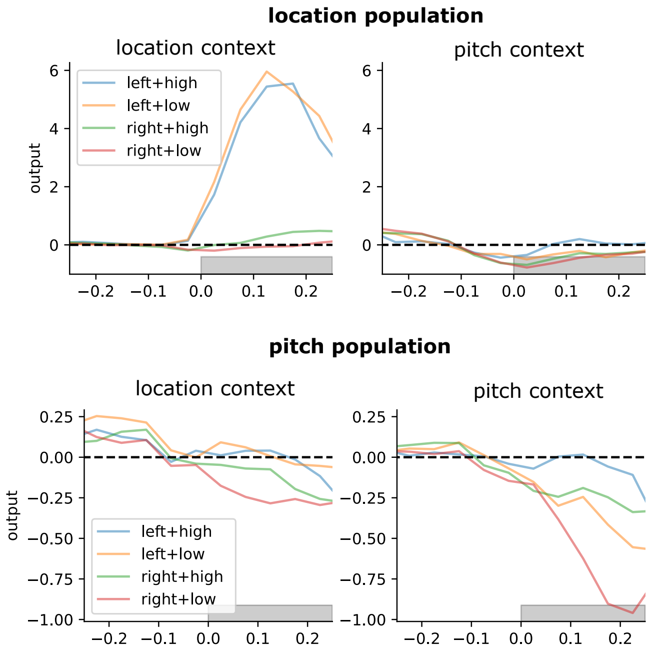

Supplementary Figure 3: *Same as Fig. 3, but separating by stimuli.* Go stimuli for the location context (left) in orange and blue. Go stimuli for the pitch context (low) in red and orange. As for other analyses, pitch is not as decodable as location (note the different y-axis).

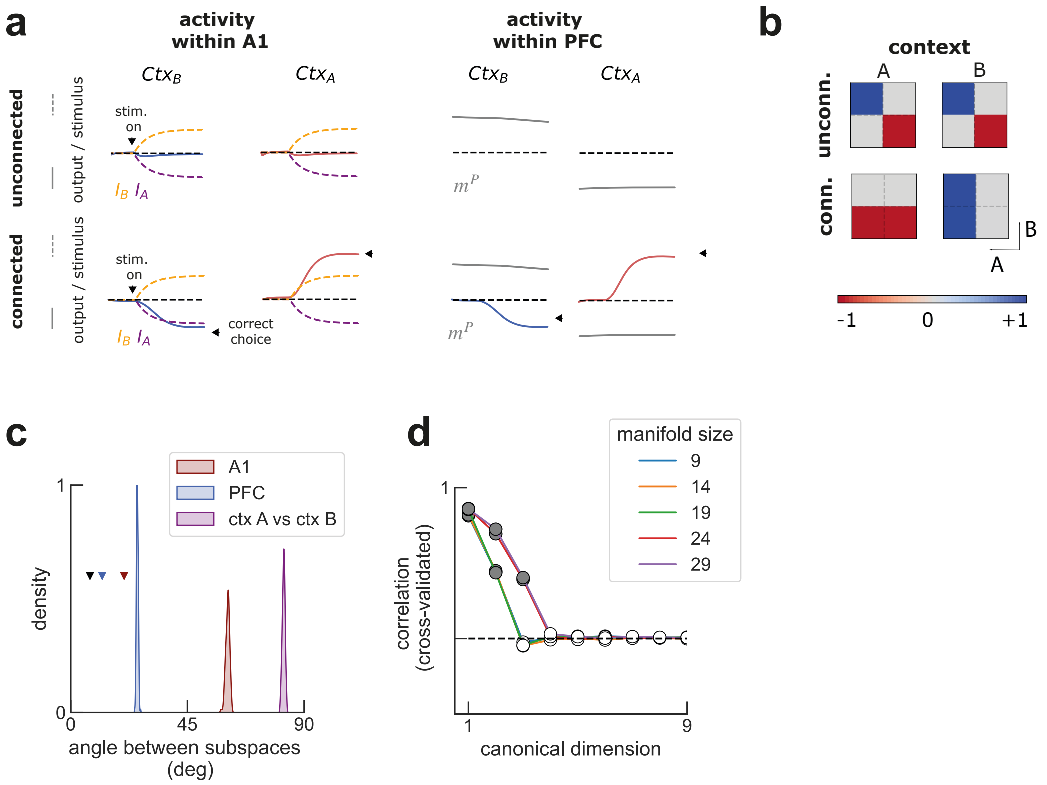

Supplementary Figure 4: *Supplementary analyses on the two-region network.* **a)** A1 and PFC in isolation did not integrate the relevant stimulus in a context-dependent fashion (unconnected), but they did when set up to cooperate through communication subspaces (connected). We found that connecting these two areas drove A1 to integrate into the recurrent dynamics the relevant, but ignore the irrelevant stimulus. Moreover, meaningful choices could be read out from PFC instead of A1 (black triangles). In the figure we illustrate two trials. Specifically, the projection of the network activity on different connectivity vectors: I_A, I_B in purple and yellow, respectively; m_A, m_p in red/blue and gray, respectively; and on the input-selector vector from A1 to PFC (Methods), red/blue. **b)** When unconnected, the two areas do not show context-dependent behavior (readout from A1), but they do so when set to interact through low-rank connectivity (readout from PFC, see also correct responses in Fig. 1a). **c)** principal angle between different subspaces (Methods). Communication subspaces inferred during opposite contexts are almost orthogonal (purple). In red and blue, the bootstrapped angle between the subspaces estimated with canonical correlation analysis and those defined by the network connectivity (Fig. 5a). For comparison, colored triangles mark the angle between connectivity subspaces and those determined by decoding context and decision from each area; black triangle marks the mean angle between the same subspaces estimated from different folds (Methods), the minimum empirical distance possible between subspaces. **d)** As described in the methods, we only used the first PCs of the neural to estimate the communication subspace using CCA. This is a typical prepossessing step (e.g. (Gallego et al. 2018). We found empirically that the estimated subspace is sensible to the number of PCs we kept (manifold size). For the purpose of Fig. 5, we only kept the first 9 PCs.

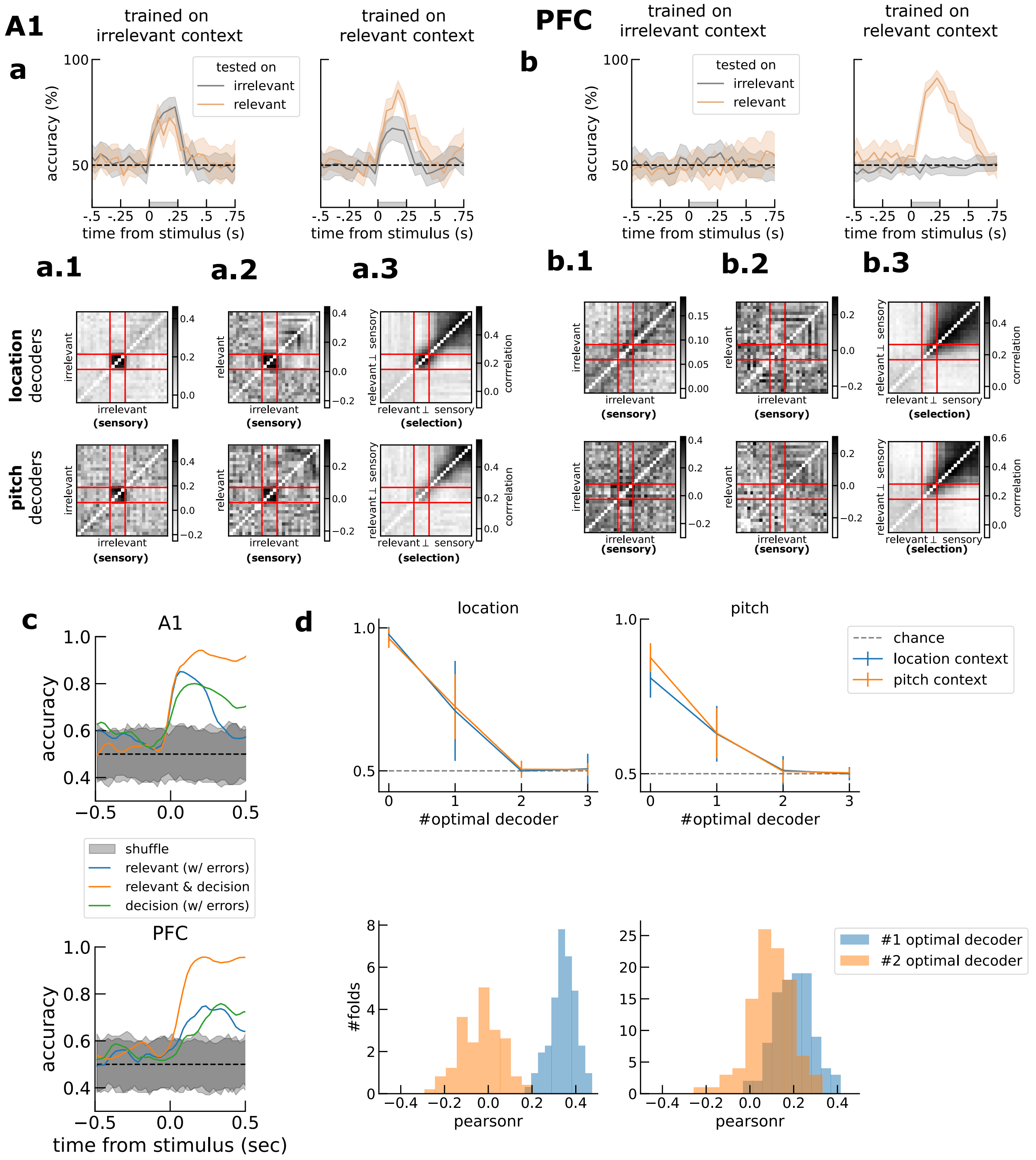

Supplementary Figure 5: a-b) Same as Fig. 1 but for pitch instead of location. Error-bars are bootstrap SEM. a.1-a.3; b.1-b.3) cross-correlation of decoding weights for different axes. In a.3, it can be seen that the relevant stimuli code has two components, one during the stimulus and another one during responses. On the other hand, for PFC (b.3), the code seems to unfold along similar codes during stimulus and response. c) Decoding analyses using error trials to decorrelate decision and relevant stimulus variables. There is above-chance decoding in both A1 and PFC, showing that there is encoding of the relevant stimulus above and beyond the encoding of choice. d) Top, Stimulus encoding dimensionality is very low, as shown by decoding from orthogonal axis to the optimal decoding. Bottom, second to optimal decoding axis is more orthogonal than the optimal.

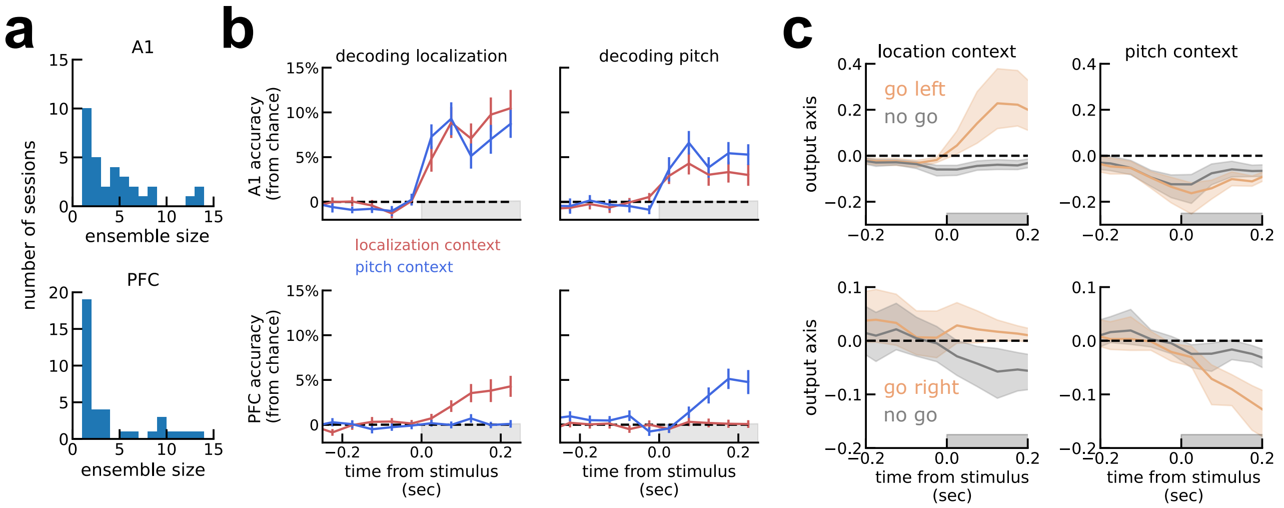

Supplementary Figure 6: Decoding from simultaneously recorded neural ensembles leads to qualitatively similar results as decoding from pseudo-trials. **a)** Size of ensembles recorded from A1 (top) and mPFC (bottom). Most sessions only have 1 recorded neuron, but some have larger size. **b)** Decoding from these small ensembles leads to qualitatively similar decoding results as Fig. S1, albeit with lower accuracy. **c)** Output gating was also qualitatively similar to pseudo-population decoding.

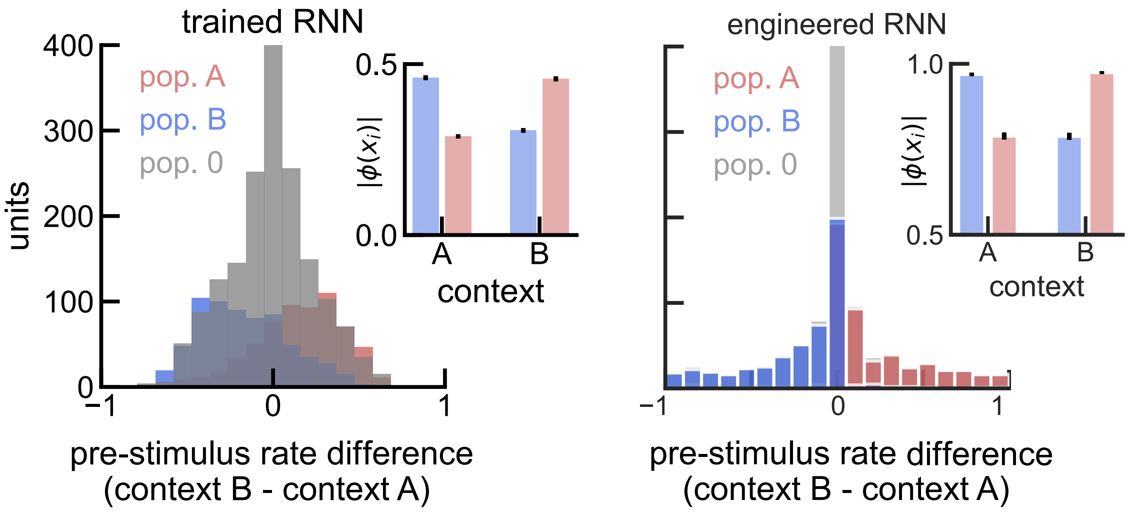

Supplementary Figure 7: Both in trained (left) and engineered RNNs (right) populations can be identified by their pre-stimulus firing rate, which is higher in opposite contexts (insets). Note that despite a discrete population structure, the distribution of pre-stimulus firing rate difference across contexts is unimodal. Compare this figure to A1 data in Fig. 3

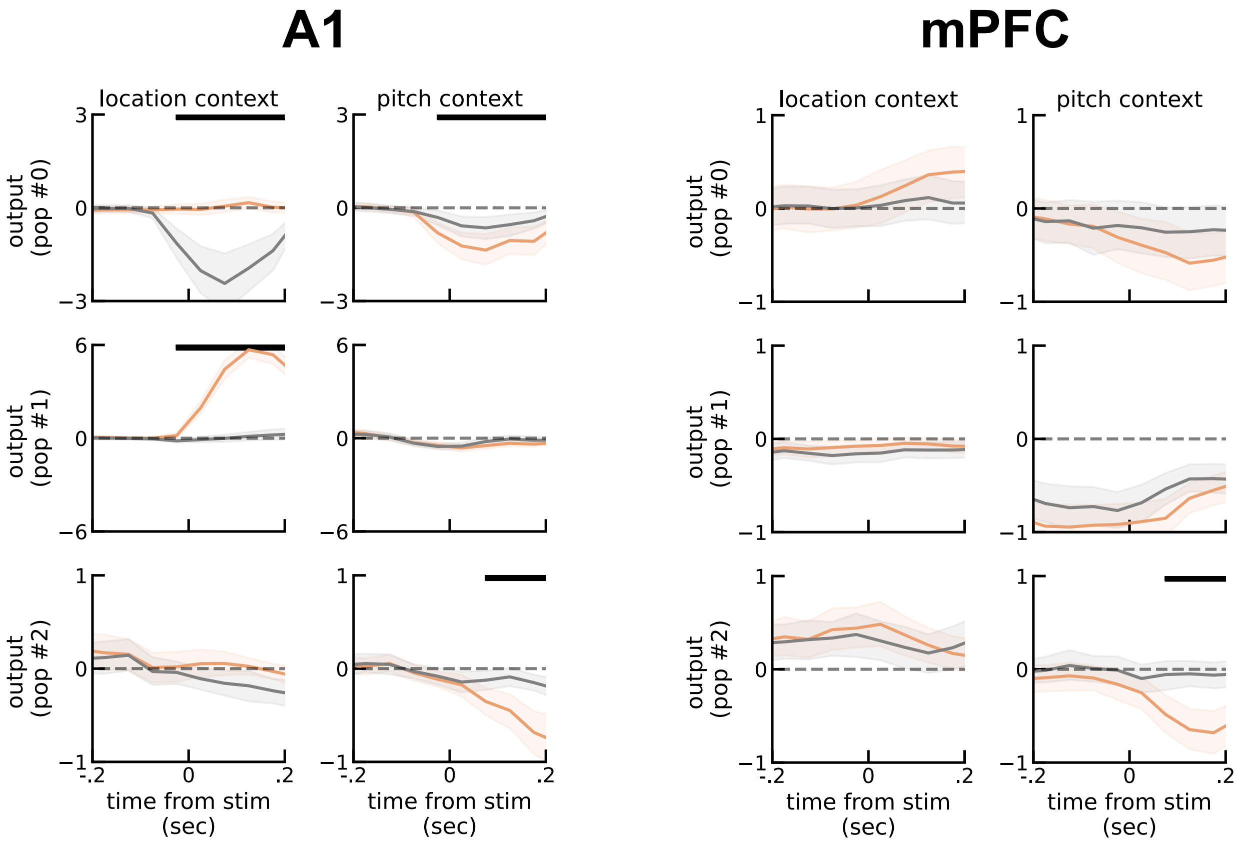

Supplementary Figure 8: Similar analyses performed in Fig. 3c for the two main population in A1, here done for all populations of A1 and PFC. See also Fig. S2a

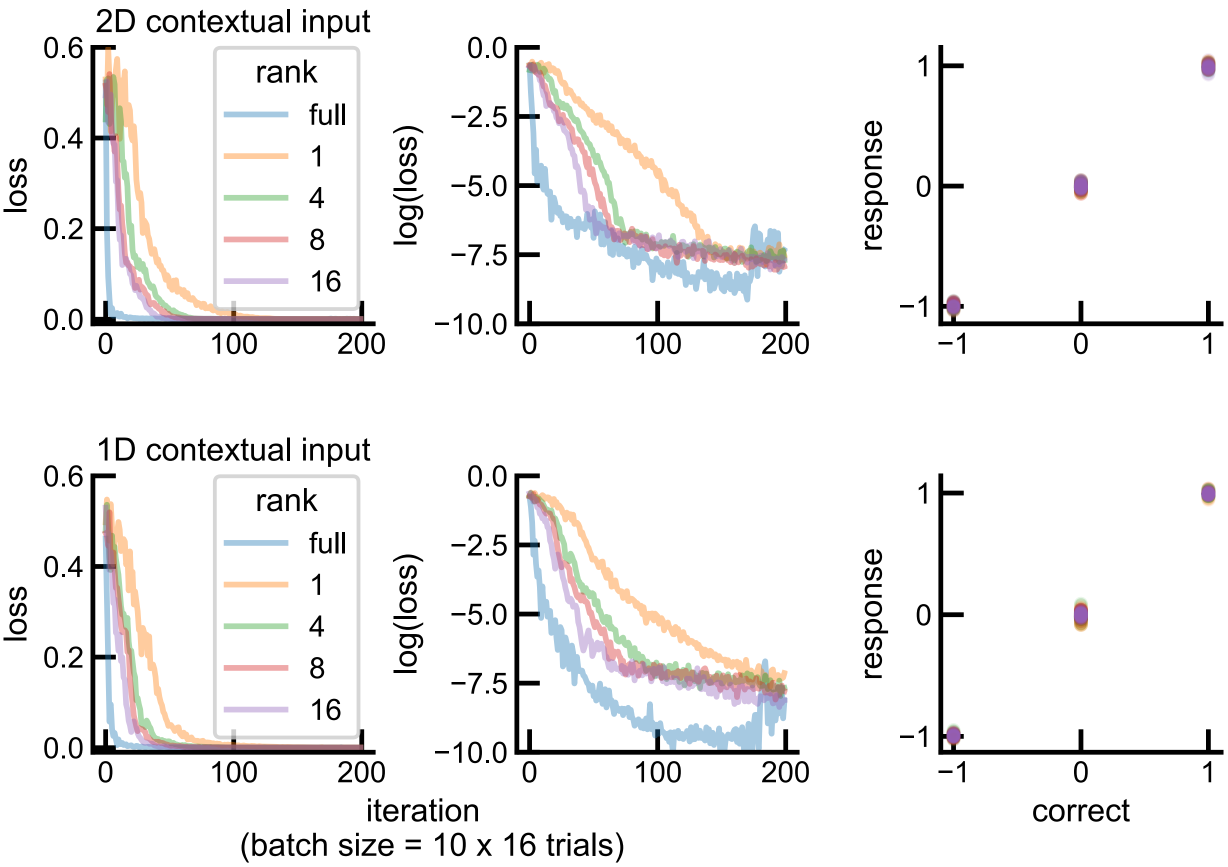

Supplementary Figure 9: A rank-1 RNN is enough to solve our context-dependent task. Allowing for higher ranks, even full rank, does not improve performance (rightmost column), but higher rank networks learn faster and full rank networks tend to over-fit. Top, network training is the same as in the main manuscript. Bottom, similar to top but contextual input is 1D (as in Fig. 5) instead of 2D.
